# Co-Injection of Sulfotyrosine Facilitates Retinal Uptake of Hyaluronic Acid Nanospheres Following Intravitreal Injection

**DOI:** 10.3390/pharmaceutics13091510

**Published:** 2021-09-18

**Authors:** Aiden Eblimit, Mustafa S. Makia, Daniel Strayve, Ryan Crane, Shannon M. Conley, Tirthankar Sinha, Ghanashyam Acharya, Muayyad R. Al-Ubaidi, Muna I. Naash

**Affiliations:** 1 Department of Biomedical Engineering, College of Engineering, University of Houston, 3517 Cullen Blvd, SERC 2009, Houston, TX 77204, USA; aeblimit@arrowheadpharma.com (A.E.); msmakia@Central.uh.edu (M.S.M.); daniel.strayve@tu.edu (D.S.); rcrane@central.uh.edu (R.C.); Tirthankar.Sinha@uth.tmc.edu (T.S.); 2 Department of Cell Biology, University of Oklahoma Health Sciences Center, Oklahoma City, OK 73104, USA; shannon-conley@ouhsc.edu; 3 Oklahoma Center for Neurosciences, University of Oklahoma Health Sciences Center, Oklahoma City, OK 73104, USA; 4 Department of Surgery, Baylor College of Medicine, Houston, TX 77030, USA; gacharya@bcm.edu; 5 College of Optometry, University of Houston, Houston, TX 77204, USA; 6 Department of Biology and Biochemistry, University of Houston, Houston, TX 77204, USA

**Keywords:** hyaluronic acid nanospheres, retina, photoreceptor, RPE, intravitreal injection, sulfotyrosine, inner limiting membrane

## Abstract

Gene and drug delivery to the retina is a critical therapeutic goal. While the majority of inherited forms of retinal degeneration affect the outer retina, specifically the photoreceptors and retinal pigment epithelium, effective targeted delivery to this region requires invasive subretinal delivery. Our goal in this work was to evaluate two innovative approaches for increasing both the persistence of delivered nanospheres and their penetration into the outer retina while using the much less invasive intravitreal delivery method. We formulated novel hyaluronic acid nanospheres (HA-NS, 250 nm and 500 nm in diameter) conjugated to fluorescent reporters and delivered them intravitreally to the adult Balb/C mouse retina. They exhibited persistence in the vitreous and along the inner limiting membrane (ILM) for up to 30 days (longest timepoint examined) but little retinal penetration. We thus evaluated the ability of the small molecule, sulfotyrosine, to disrupt the ILM, and found that 3.2 µg/µL sulfotyrosine led to significant improvement in delivery to the outer retina following intravitreal injections without causing retinal inflammation, degeneration, or loss of function. Co-delivery of sulfotyrosine and HA-NS led to robust improvements in penetration of HA-NS into the retina and accumulation along the interface between the photoreceptors and the retinal pigment epithelium. These exciting findings suggest that sulfotyrosine and HA-NS may be an effective strategy for outer retinal targeting after intravitreal injection.

## 1. Introduction

The vast majority of defects underlying inherited retinal degeneration (IRD) in humans result from mutations in genes expressed in photoreceptors and the retinal pigment epithelia (RPE) [1]. As a result, in the past decades much effort has been focused on the development of novel delivery methods for targeted therapeutic delivery to the outer retina. Nanoscale delivery of genetic therapies to ameliorate phenotypes associated with IRD has taken many forms, including gene supplementation, knockdown, and most recently, delivery of CRISPR based approaches for gene editing (recently reviewed in [2]). While all of these approaches have been successfully employed in animal models, only one retinal gene therapy has thus far been FDA-approved for use in humans, an adeno-associated virus (AAV) carrying RPE65, which is subretinally delivered to patients with Leber’s congenital amaurosis (first described in [3,4,5]).

In spite of the success of AAV-mediated RPE65 gene therapy, several limitations have hampered more widespread application of retinal gene therapy, including the need to use viral compaction methods (AAV) for gene delivery and the need to use invasive subretinal injection to target the outer retina. While AAVs have a good safety profile, they have limited carrying capacity and viral tropisms that can affect cellular uptake. Much effort has gone into further AAV development, but there has also been great interest in developing non-viral gene formulation/compaction approaches. A number of nanoparticles have been evaluated in the retina [6,7]. One of the most well-characterized examples has been polyethylene glycol-linked polylysine (CK30PEG) nanoparticles which do not cause adverse effects in the retina and have a large carrying capacity (tested up to 14 kbp in the retina and 20 kbp in the lung [8,9,10,11]). These nanoparticles exhibited persistent gene expression and phenotypic rescue in animal models [9,12,13]; however, in common with other gene therapy approaches, phenotypic rescue was incomplete, and efficient outer retinal targeting required subretinal injections.

Subretinal injections cause retinal detachment, can trigger inflammatory responses, and can lead to recruitment of immune cells into the subretinal space [6]. These risks are considered critical limitations for subretinal injection during clinical trials [14], and development of alternative delivery approaches is essential. In contrast, intravitreal injection is much less invasive, and is widely used to target the inner retina in animal models, including retinal ganglion cells as targets for Leber hereditary optic neuropathy [15], Müller cells for delivery of neurotrophic factors [16], and bipolar and amacrine cells for optogenetic therapy [17]. Intravitreal injection is also in common clinical use, for example, for delivery of anti-VEGF agents used to treat neovascular age-related macular degeneration. However, while some studies have demonstrated transretinal movement of intravitreally delivered nanoparticles [18], the majority of studies have shown that delivery of nanosized particles carrying drugs or genetic cargo into the outer retina after intravitreal injection remains extremely difficult (reviewed in [19]).

A key barrier to retinal uptake after intravitreal injection is the presence of the vitreous and the inner limiting membrane (ILM). The vitreous is a transparent gel composed of collagen fibrils and glycosaminoglycans such as hyaluronic acid, and can hamper the mobility of intravitreally delivered material, preventing it from reaching the retina. The ILM is at the vitreoretinal interface and forms the basement membrane of the Müller glia. In common with other basement membranes, it is composed of a collagen IV network linked with proteoglycans, laminin, and fibronectin [20,21]. The ILM functions as a barrier that can impede the transfer of nanocarriers into the retina [20].

To help overcome these barriers and promote the diffusion of nanocarriers into the retina after intravitreal delivery, an innovative new strategy is proposed, wherein co-delivery of cross-linked hyaluronic acid (HA), nanospheres (HA-NS), and sulfotyrosine (ST) enhanced the diffusion of HA-NS into the outer retina. Considering the high concentration of HA in the vitreous humor, we used HA-NS to minimize adverse immune or inflammatory reactions in the vitreous after their intravitreal injection. To help improve penetration of the retina, we evaluate the benefits of co-delivery of HA-NS with sulfotyrosine (ST), a compound designed to transiently open the ILM. Our findings demonstrate that ST and HA-NS are safe for use in the retina, that HA-NS are persistent in the eye, and that co-delivery of low-dose ST dramatically increases penetration of HA-NS in the outer retina.

## 2. Materials and Methods

### 2.1. Animals

Adult albino Balb/C mice (P30–P90) were used in this study. All experiments involving mice were approved by the local institutional animal care and use committee at the University of Houston, and adhered to the recommendations in the Guide for the Care and Use of Laboratory Animals of the National Institutes of Health and the Association for Research in Vision and Ophthalmology on the use of animals in research. Animals were bred in-house and reared under cyclic lighting conditions (12 h light/dark, ∼30 lux). Mice were euthanized using CO_2_ asphyxiation followed by decapitation, and then the eyes were harvested and used as indicated below.

### 2.2. Fabrication of Fluorescent Hyaluronic Acid Nanospheres

HA-NSs were fabricated, similar to our previous research, using a modified hydrogel template strategy [22,23]. To fabricate the HA-NS-250 nm or the HA-NS-500 nm, first an ethyl cellulose template for each was prepared by using a polydimethylsiloxane (PDMS) template containing 250 nm or 500 nm posts, respectively. Onto this PDMS template, ethyl cellulose solution in ethanol (5%, 5 mL) was transferred and evenly spread. The ethyl cellulose solution was then allowed to dry (~2 h) at room temperature. After complete drying, the ethyl cellulose template was peeled off from the PDMS template. Thus, fabricated ethyl cellulose template contained circular wells of 250 nm or 500 nm diameter and depth, respectively. A 5% solution (10 mL) of hyaluronic acid methacrylate (MW 20,000–30,000, Millipore Sigma, St. Louis, MO, USA) was mixed with fluorescein-O-methacrylate (5 mg, Millipore Sigma) and was used to fill the ethyl cellulose templates. After filling the ethyl cellulose, the template was then subjected to UV light (30 min) to photo-crosslink the hyaluronic acid methacrylate. The ethyl cellulose templates were dissolved in ethanol to free the NSs. The ethanol solution was centrifuged to pellet the NSs. The pellet was resuspended in ethanol and washed several times to completely remove the ethyl cellulose. Finally, the ethanol solution containing HA-NSs was filtered through 1 µm filters to obtain the HA-NSs. A similar procedure was followed wherein a solution hyaluronic acid methacrylate (10 mL, 5% in H_2_O) and acryloxyethyl thiocarbamoyl rhodamine-B (5 mg, Millipore Sigma) was used to fabricate the HA-NS-500 to include rhodamine.

To calculate the concentration of nanospheres in the original suspension, 1 µL of HA-NS suspension was diluted 20-fold in saline, 10 µL of this diluted sample was loaded on a slide and covered with a 2.2 cm square cover slip. The 10 µL of HA-NSs was evenly and fully distributed under the coverslip. At 20× magnification, the image under the microscope is divided into 10 horizontal and 10 vertical boxes and the number of NSs is counted in each box. The area of 20× image is calculated and multiplied by a factor abstained from division of area of cover slip by area of 20× image, then final concentration of nanospheres in each µL suspension is reported: 60/column × 10 column ÷ (320 µm × 320 µm) × 22 µm × 22 µm × 10^6^ ÷ 10 µL × 20 times dilution factor = 5.8 × 10^6^ nanospheres/uL suspension. Nanosphere size was measured via dynamic light scattering using a Malvern Zetasizer (Nanoseries, Malvern, Worcestershire, UK) instrument. At least three separate preparations for each size were analyzed.

### 2.3. High Performance Liquid Chromatography (HPLC) Measurements

The fluorescence intensity of the HA-NS-250 was assessed by HPLC. First, different concentrations (5, 10, 20, 30, 40, and 50 µg/µL) of free fluorescein were used to generate a standard curve based on the area under the curve as calculated by the Breeze 2 software (Waters, Milford, MA, USA). Different concentrations of standards were injected and quantified for linearity. A processing method was generated using identified retention times and standard curve plotted for each component, integrated to auto-identify the peaks and auto-calculate the concentration of the respective compounds. The mobile phase was saline (70%) and acetonitrile (30%) in isocratic method. A 5 µL volume of HA-NS-250 stock was diluted 20 fold in PBS to a final volume of 100 µL and then centrifuged at 18,000× *g* for 15 min to fully pellet the NSs and the supernatant was removed. The HA-NS-250 pellet was resuspended in 100 µL PBS. Free fluorescein was prepared in PBS and 50 µL of each sample was subjected to HPLC analysis. The HPLC setup was composed of a Waters binary HPLC pump (1525), Waters auto-sampler (2707), Waters multi wavelength fluorescence detector (2475), and a Waters X-Bridge C18 3.5 μm column with dimensions of 4.6 × 250 mm (Waters, Milford, MA, USA).

### 2.4. Preparation of Sulfotyrosine Solution

H-Tyr(SO_3_H)-OH sodium salt (sulfotyrosine-ST) was purchased from BACHEM (Torrance, CA, USA, Cat#4033889, Lot#1056704). The sulfotyrosine sodium salt was weighed out and dissolved in endotoxin-free 0.9% saline at the desired concentrations at 70 °C. Working solutions of ST (prepared from the dissolved sulfotyrosine sodium salt) were prepared and mixed with equal volumes of nanospheres by tapping gently, immediately before injections. As controls, equal volumes of nanospheres were mixed with saline prior to injection.

### 2.5. Lipopolysaccharide (LPS)

LPS was purchased from Sigma (L2262-5MG) and reconstructed in 0.9% saline at the concentration of 2 µg/µL.

#### 2.5.1. Intravitreal and Subretinal Injection

Intravitreal and subretinal injections were performed as previously described [9,24,25]. Within the sterile surgical room, adult Balb/c mice were anesthetized by an intraperitoneal injection of 85 mg/kg ketamine and 14 mg/kg xylazine (Henry Schein Animal Health, Dublin, OH, USA). Eyes were dilated with 1% cyclopentolate (Bausch and Lomb, Rochester, NY, USA). For intravitreal injections, a very shallow puncture was made through the sclera in the dorsal hemisphere using a beveled 30-gauge needle (BD Biosciences, Franklin Lakes, NJ, USA). Through this puncture, a 33-gauge blunt-end needle was introduced into the vitreous cavity and 1.5–3 µL of material (nanospheres, saline, LPS, ST, or ST/HA-NS mixture) was injected into the vitreous space near optic nerve using a 5 µL microinjection Syringe (Hamilton, Reno, NV, USA). For subretinal injections, the needle was then advanced gently through the retina and into the subretinal space, where the nanosphere suspension was gently delivered. Visualization of a subretinal bleb during injection and subsequent fundoscopic and OCT imaging confirmed successful subretinal delivery. Triple antibiotic ointment (Taro Pharmaceuticals, Inc., Hawthorne, NY, USA) was applied to each eye after injection.

#### 2.5.2. Electroretinography, Fundus Imaging, and Optical Coherence Tomography (OCT)

Full-field electroretinograms (ERGs) were recorded as described previously [26]. Briefly, mice were dark-adapted overnight and were anesthetized using 85 mg/kg ketamine and 14 mg/kg xylazine (Henry Schein Animal Health, Dublin, OH, USA). Eyes were dilated with 1% cyclopentolate and covered in Gonak (Akorn Pharmaceuticals, Lake Forest, IL, USA). Platinum wire loops were placed in contact with the cornea through a layer of Gonak. Using the UTAS system (LKC, Gaithersburg, MD, USA) full-field scotopic ERG responses were recorded from each eye in response to a single 157-cd s/m^2^ flash. Fundus imaging was performed using the Micron IV system (Phoenix Research Laboratories, Pleasanton, CA, USA). Animals were anaesthetized/dilated as for ERG but were not dark-adapted. Brightfield images were captured first, and then HA-NS distribution was analyzed by imaging with the green filters (451.5–486.5 nm excitation and 488 nm emission, for fluorescein) or red filters (553 nm excitation and 627 nm emission). All images were captured using StreamPix^®^ software (Phoenix Research Laboratories, Pleasanton, CA, USA). For OCT, animals were anesthetized and eyes dilated as for fundus imaging. OCT images were captured using an Image Guided OCT2 (Phoenix Research Laboratories). Images were captured at 100 fpm using the Reveal OCT software.

#### 2.5.3. Fluorescence Imaging and Counting HA-NS in the Retina

HA-NS distribution in the mouse retina was analyzed as previously described [9]. Briefly, the whole eye was enucleated, cryoprotected and embedded without fixation. Eyes were cryosectioned along the nasal-temporal plane from superior to inferior and 20 µm thick sections were collected every ~200 µm. Sections were post-fixed in 4% paraformaldehyde for 5 min, washed in 1 × PBS, stained with DAPI (Cat# 62248, ThermoFisher, Waltham, MA, USA, 1:1000 dilution) for 10 min to counterstain nuclei, then washed three times for five min in 1 × PBS. Slides were mounted in ProLong gold anti-fade mounting media (ThermoFisher). Images were collected at 20× (0.8 air) using a Zeiss Confocal Microscope (Zeiss, Jena, Germany). Full section views were generated by tiling 20× images using the Zen 3.2 Blue software. Surface plots were generated using ImageJ https://imagej.nih.gov/ij/plugins/surface-plot-3d.html (accessed 17 September 2021). For the green channel used to image the HA-NS-250, GFP filters were used with the excitation peak at 488 nm, and emission peak at 509 nm. For the red channel used to image the HA-NS-500, the filters have an excitation peak at 553 nm and an emission peak at 627 nm.

To generate graphical maps estimating HA-NS distribution in the retina, images were captured from 20 µm sections collected ~200 µm apart (corresponding to rows 1–10 on maps). Images were captured from adjacent frames from each section from temporal to nasal (corresponding to columns a–j on maps). Counting was done in ImageJ. HA-NS around ILM and in the vitreous were not counted; only HA-NS fluorescent puncta in the retina were counted. In cases where the fluorescent spot in the retina was large (i.e., likely more than one HA-NS), the fluorescence intensity of the large/aggregate spot was divided by the intensity of a single HA-NS puncta to estimate of how many HA-NS were in the aggregate. In graphical maps, white squares represent images containing fewer than 10 HA-NS-250 puncta, green squares contained 10–199, yellow squares contained 200–499, orange squares 500–999, and red squares ≥1000. In order to estimate the total number of HA-NSs in whole retina, we multiplied number of NSs obtained from one slide (each slide contains 10 retinal sections from throughout the whole retina (i.e., rows 1–10 on graphical maps) by 10 (to account for the sections skipped in the 200 µm between the sections that were collected and counted).

#### 2.5.4. H&E Staining and Photoreceptor Nuclei Counts

Eyes were enucleated and fixed overnight with Davidson’s Fixative (32% ethanol, 11% acetic acid, 2% formaldehyde). After paraffin embedding as described previously [27], 7 µm sections were cut using a microtome (Leica Biosystems, Wetzlar, Germany). Slides were deparaffinized and used for H&E staining using standard protocols. Images were captured using a Zeiss Axioskop equipped with a Zeiss Axiocam (Zeiss) using a 20× (NA: 0.45 air) or 100× (NA: 1.30 oil) objective. Images were captured at 500 µm intervals migrating peripherally (both inferior and superior) from the optic nerve head. Images were analyzed using ImageJ software. For the nuclear count, nuclei were enumerated in 100 μm-wide portions of the retina centered at the indicated distances from the optic nerve, and all nuclei in the outer nuclear layer within this area were counted using the ImageJ cell counter plugin.

#### 2.5.5. Immunofluorescence Labeling

Immunofluorescence labeling for laminin was performed on paraffin embedded sections. After deparaffinization, antigen retrieval was performed by boiling sections in 0.01 M Tris, EDTA buffer (pH 9.0) for 30 min, followed by cooling for 30 min. Slides were washed in PBS, incubated for 1 h at room temperature in hybridization buffer (10% normal goat serum, 0.1% Triton X-100, PBS), then incubated overnight in primary antibody (rabbit anti-laminin, Cat# L9393, RRID:AB_477163 Sigma, 1:100 dilution) diluted in hybridization buffer. Slides were then washed in PBS, incubated with secondary antibody diluted in hybridization buffer at room temperature for 2 h, washed in PBS, mounted with anti-fade medium (Prolong; ThermoFisher) to reduce bleaching, and coverslipped. For F4/80 immunolabeling, frozen retinal sections were used as previously described [28], and the labeling protocol was performed as described above but without antigen retrieval. Rat anti F4/80 antibody was used at a dilution of 1:500 (Cat# MCA497RT, RRID# AB_1102558, Bio-Rad, Temecula, CA, USA). Secondary antibodies were goat anti-rat or goat anti-rabbit Alexa Fluor 555 (Cat# A21434 and Cat# A21428, Thermo Fisher). All immunostaining experiments were performed independently using three biological replicates. Fluorescent images were captured with a Zeiss Confocal Microscope (Zeiss), at 20× and figures showing of laminin and F4/80 labeling are single plane confocal images.

### 2.6. Electron Microscopy (EM)

Eyes were harvested, fixed, and processed for transmission EM analysis as described previously [24]. Images of the inner retina were captured at 25,000×.

#### Cytokine Protein Assay

Mouse eyes were enucleated, snap frozen in liquid nitrogen, and pulverized. A total of 500 μL of 1× PBS containing a protease inhibitor cocktail was added to the pulverized tissue powder. The samples were homogenized using a pestle pellet homogenizer and freeze–thawed twice followed by centrifugation for 10 min at 13,000 rpm at 4 °C. Supernatants were evaluated by BCA protein concentration assay and kept frozen at −80 °C overnight. Cytokines (IL-6, IL-1β, TNF-α, and IFN-γ) were measured using a mouse-specific V-PLEX Proinflammatory Panel using the electrochemiluminescence multiplex system Sector 2400 imager (MesoScale Discovery System, Cat# K15048G). *n* = 3 eyes per group.

## 3. Statistical Analysis

All data are plotted as the mean ± SD using Graphpad Prism 8.4.3. ERG data were analyzed by one-way ANOVA with Tukey’s post hoc comparison. For cytokine protein analysis and HA-NS-250 counts, differences between groups were analyzed by one-way ANOVA with Tukey’s post hoc comparison. For spidergrams, differences between groups were assessed using two-way ANOVA with Tukey’s post hoc comparison. Significance was set at *p* < 0.05. Throughout the manuscript * *p* < 0.05, ** *p* < 0.01, *** *p* < 0.001, and **** *p* < 0.0001 for indicated pairwise comparisons.

## 4. Results

### 4.1. Characterization of Hyaluronic Acid Nanospheres

**Figure 1:**
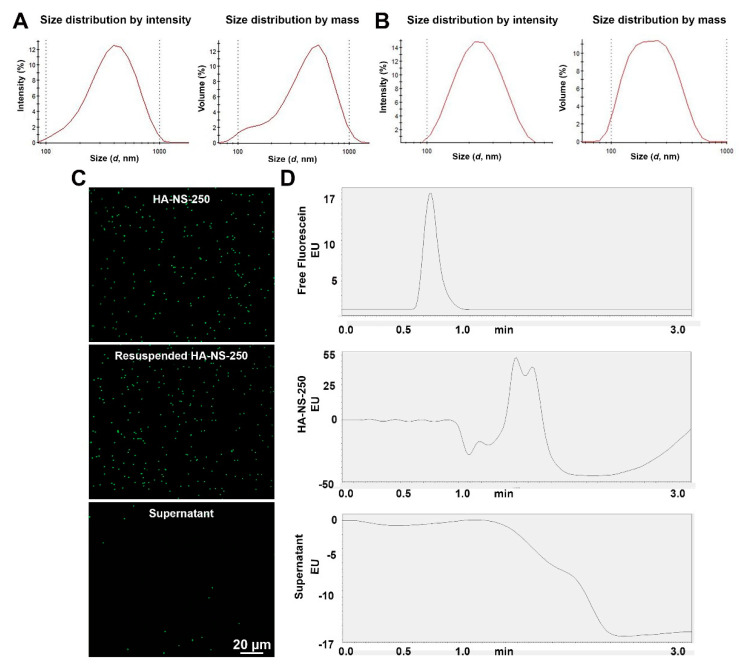
Characteristics of fluorescein-conjugated HA-NS-250. (**A**,**B**) Dynamic light scattering was used to characterize the size of the HA-NS-500 (**A**), and HA-NS-250 (**B**). (**C**) Nanospheres were diluted in saline and imaged under a fluorescence microscope (top image) after being pelleted and resuspended (middle image), no significant aggregation is observed, and very little fluorescein is detected in the supernatant (bottom image). (**D**) To confirm that fluorescein is not being released from NS, fluorescein (positive control, top), resuspended HA-NS-250 (middle), and supernatant (bottom) from pelleted HA-NS-250 underwent HPLC. Shown are HPLC traces; no signal is detected in supernatant. EU: emission unit. Scale bar 20 µm.

Hyaluronic acid (HA) was chosen to formulate the nanospheres (NS, here referred to as HA-NS) used in this study as it is a non-sulfated linear polysaccharide that is biodegradable, biocompatible, nontoxic, hydrophilic, and non-immunogenic, and it is abundantly present in the vitreous. HA also contains reactive functional groups (-COOH and -OH) to which targeting ligands can be coupled to develop targeted delivery systems. In this study, we fabricated the cross-linked HA-nanospheres using photocrosslinkable hyaluronic acid methacrylate. HA-NS can be formulated at various sizes and coupled with various cargoes; in this study, we have evaluated 250 nm NS (HA-NS-250) and 500 nm NS (HA-NS-500). To help assess distribution of the NS, hyaluronic acid methacrylate was mixed with fluorescein-O-methacrylate to fabricate green fluorescent HA-NS-250, and hyaluronic acid methacrylate was mixed with methacryloxyethyl thiocarbamoyl rhodamine-B followed by photopolymerization to fabricate red fluorescent HA-NS-500. To confirm the predicted sizes and size distributions of the NS, we performed DLS measurements on three independent samples of particles (Figure 1A,B show size distributions for HA-NS-500 [A] and HA-NS-250 [B]). Mean NS size was consistent across samples, with HA-NS-250 exhibiting a mean size of 259.2 ± 8.5 nm, and NS-500 exhibiting a mean size of 418.9 ± 4.1 nm.

To confirm that the fluorescein was fully attached to the NS (thus enabling us to use fluorescein as an indicator of the presence of NS post-injection), we evaluated whether fluorescein was released from HA-NS-250 in suspension. HA-NS-250 were pelleted by centrifugation, the supernatant was removed, and the pellet was resuspended. Fluorescence microscopy demonstrates that discrete HA-NS-250 are easily visualized in both the original HA-NS suspension and the re-suspended pellet. We observe no signs of aggregation of the HA-NS-250 after centrifugation, and very little green signal in the supernatant (Figure 1C). To help confirm these findings, we used HPLC as an alternative method to evaluate fluorescence. A fluorescein peak was detected in both the free fluorescein positive control (Figure 1D, top), and the resuspended HA-NS-250 (Figure 1D, middle), but not the supernatant from pelleted HA-NS-250 (Figure 1D, bottom), confirming that the fluorescein does not detach from the nanospheres. To determine the stability of HA-NS-250 during storage at 4 °C, the number of nanospheres was counted shortly after formulation and then again four months later. There was a negligible difference during this period; the initial concentration was 5.8 × 10^6^ HA-NS-250/μL after formulation and was 5.6 × 10^6^ HA-NS-250/µL of suspension after four months of storage. These findings suggest that HA-NS-250 are stable, do not aggregate with centrifugation and remain tightly linked to fluorescein, making them suitable for subsequent evaluation.

### 4.2. HA-NS Exhibit Persistence in the Vitreous but Low Retinal Penetration after Intravitreal Injection

**Figure 2:**
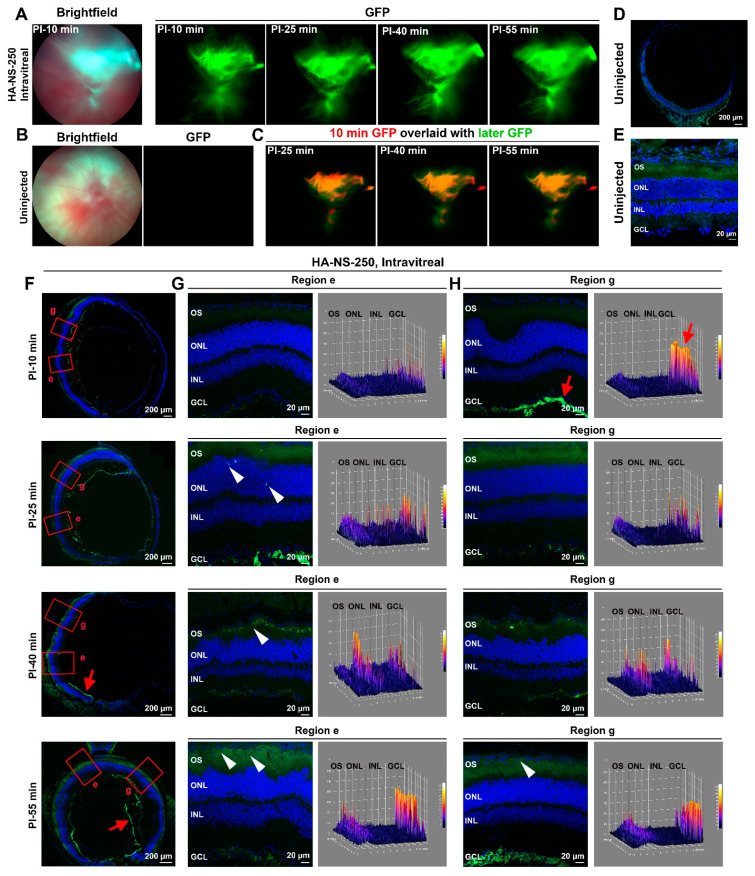
Short-term distribution of HA-NS-250 after intravitreal injection. Adult Balb/C mouse eyes were intravitreally injected with 2 µL of HA-NS-250 (5.8 × 10^6^ HA-NS-250/µL). (**A**,**B**) Fundus images were taken using white light (left panel, (**A**,**B**) or GFP filters (all other panels in (**A**,**B**)) at time points ranging from PI-10 min to PI-55 min. (**C**) To observe how distribution of the HA-NS-250 signal changes over time, the PI-10 min image is shown pseudocolored red with subsequently captured images (PI-25, 40 and 55 min) overlaid in green. (**D**,**E**) Shown are representative images from uninjected control eyes. (**F**–**H**) Injected eyes were harvested at the indicated timepoints and serially cryosectioned (*n* = 3). (**F**) Shown are representative sections with HA-NS-250 fluorescence shown in green and nuclei counterstained with DAPI in blue. Red boxes highlight regions shown in (**G**,**H**). (**G**,**H**) On the left are representative regions from the selected sections. On the right are surface plots showing the intensity of green signal throughout the section. Red arrows highlight HA-NS-250 accumulating in the vitreous adjacent to the ILM. Arrowheads highlight HA-NS-250 in the retina. OS: outer segment, ONL: outer nuclear layer, INL: inner nuclear layer, GCL: ganglion cell layer. Scale bars: 200 µm (**D**,**F**) and 20 µm (**E**,**G**,**H**).

To begin our examination of the utility of HA-NS for retinal delivery, we intravitreally injected 2 µL of HA-NS-250 (at 5.8 × 10^6^ HA-NS-250/µL) into the adult Balb/C mouse retina and tracked fluorescein distribution in the hour following injection. Figure 2A shows fundus images of the same mouse captured at 10 min, 25 min, 40 min, and 55 min post injection (PI). To help evaluate the extent to which the fluorescent HA-NS-250 gradually diffused across the eye, we overlaid the image captured at 10 min (Figure 2C, pseudocolored red) with those taken at later timepoints (Figure 2C, green). The fluorescent area expands slightly within the first hour, but the pattern remains fairly constant throughout that time period. The fluorescence signal is largely concentrated in the central retina around the optic nerve, which is consistent with the injection site. No fluorescent signal is observed in the uninjected control eye (Figure 2B).

To help understand the distribution of the HA-NS-250 in the retina during this early time post-injection, eyes were collected from animals at the same timepoints highlighted in Figure 2A. The unfixed tissue was frozen and sections were collected evenly throughout the eye (representative example sections shown in Figure 2F), and adjacent images were evaluated from each section (representative example images shown in Figure 2G,H). At all timepoints, the large majority of the HA-NS-250 fluorescence was found in the vitreous space, often adjacent to the ILM.

As can be seen in the top row of Figure 2F–H, within the first 10 min PI, HA-NS-250 are mainly localized in the vitreous adjacent to the ILM (red arrow, Figure 2H) with little detectable signal in the retina. The right panels in Figure 2G,H are heat maps representing green intensity across the retinal layers. At all timepoints, there is significant autofluorescence in the outer segment layer, (including in uninjected controls, Figure 2D,E). This autofluorescent background varies in intensity from eye to eye, but is characterized by a diffuse green signal throughout the outer segment layer, in contrast to the signal arising from the HA-NS-250 which is punctate in nature. A similar pattern is seen at PI-25 min (Figure 2F–H, second row) with the majority of the HA-NS-250 in the vitreous, and some HA-NS-250 detected within the retina (white arrowheads), including in the outer nuclear layer. By PI-40 and PI-55 min, some additional punctate HA-NS-250 signal is found in the retina in the region between the outer segment layer and the RPE (Figure 2F–H, third and fourth row, arrowheads highlight punctate HA-NS-250 to help distinguish from background autofluorescence). However, at all of these timepoints, the majority of HA-NS-250 remains in the vitreous adjacent to the ILM.

**Figure 3:**
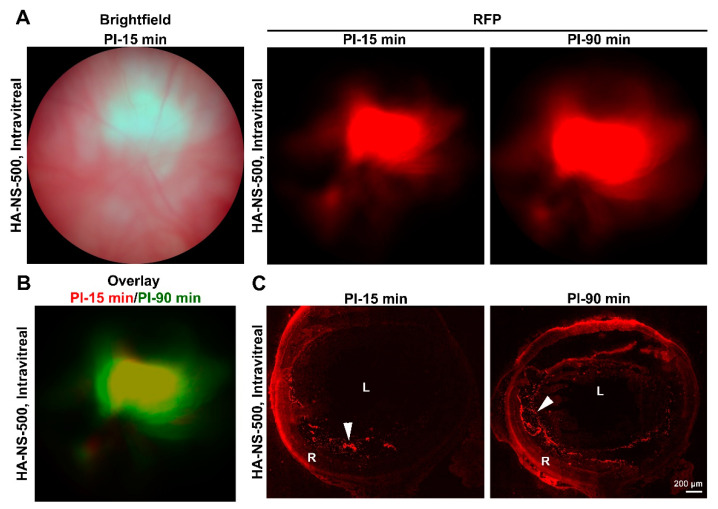
Short-term distribution of HA-NS-500 after intravitreal injection. Adult Balb/C mice were intravitreally injected with HA-NS-500 (2 µL at 5.0 × 10^6^ HA-NS-500/µL). (**A**) Left: Shown is a brightfield fundus image captured at PI-15 min. Right: Shown are fundus images captured using RFP filters at 15 and 90 min post-injection. (**B**) To observe how distribution of the HA-NS-500 signal changes over time, the PI-15 min image is shown in red with the PI-90 min image overlaid in pseudocolored green. (**C**) Injected eyes were harvested at the indicated timepoints and serially cryosectioned. Shown are representative sections with HA-NS-500 fluorescence shown in red. Arrowheads highlight accumulation of HA-NS-500 between the lens and retina, and along the inner limiting membrane. L: lens, R: retina Scale bar: 200 µm.

We next conducted a similar early stage analysis of the distribution of the larger HA-NS-500 after intravitreal injection into the adult mouse retina (Figure 3, 2 µL injection at 5.0 × 10^6^ HA-NS-500/µL). Rhodamine fluorescence was easily visible by fundus imaging at PI-15 min and PI-90 min (Figure 3A). The distribution of HA-NS-500 expanded slightly from 15–90 min (overlay image shown in in Figure 3B), but the overall distribution was similar at the two timepoints. Similar to the case with HA-NS-250, evaluation of retinal cross-sections (Figure 3C), showed that the vast majority of HA-NS-500 accumulated in the vitreous (arrowheads), mostly along the ILM and the edge of the lens, with little or no penetration into the retina. Combined, these findings demonstrate that at all timepoints examined within the early hours after intravitreal injection, the majority of HA-NS-250 and HA-NS-500 do not penetrate into the retina but remain in the vitreous, mostly concentrated at the injection site.

**Figure 4:**
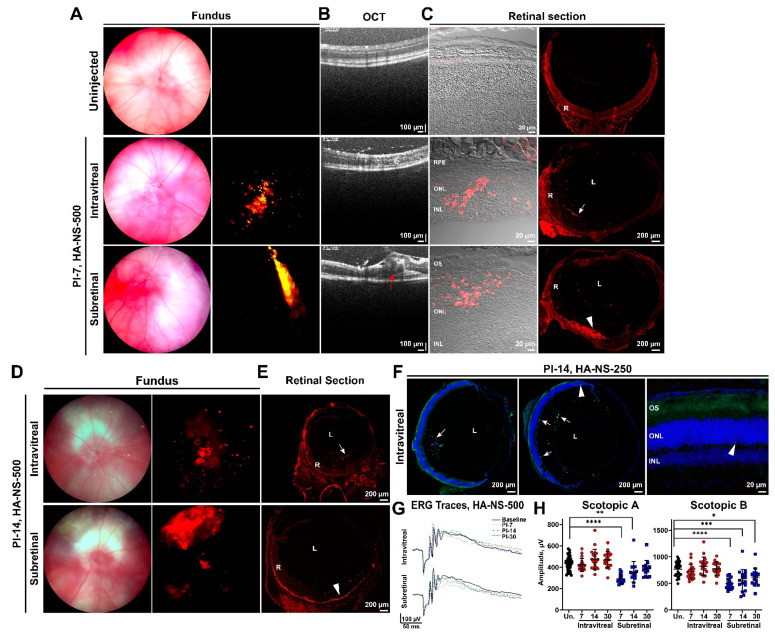
Intravitreal injection does not lead to wide distribution of HA-NS-500 or HA-NS-250 in the retina. (**A**–**E**) HA-NS-500 were injected either intravitreally or subretinally into the adult mouse retina. Follow up was performed at PI-7 days (**A**–**C**), or PI-14 days (**D**,**E**). (**A**,**D**) Shown are representative brightfield (left) and RFP (right) fundus images. (**B**) In vivo retinal cross sections were captured via OCT in the region of the injection. Red arrow highlights retinal disruption in subretinally injected eyes. (**C**) Tissues were harvested and cryosectioned. Left panel shows native rhodamine fluorescence (red) within localized areas of the retina. Middle panel shows native rhodamine fluorescence in whole retinal cross section, arrows highlight accumulation of HA-NS-500 along the ILM, arrowheads highlight accumulation in the subretinal space. (**D**) Shown are representative brightfield and RFP fundus images (as in (**A**)) captured at PI 14 days. (**E**) Shown are representative retinal cross sections with native red signal (as in (**C**)). Arrows show accumulation of HA-NS-500. (**F**) HA-NS-250 were injected and tissues harvested at PI-14 days. Shown are native fluorescein fluorescence (green) and DAPI (blue). Arrows highlight accumulation of HA-NS-250 in the vitreous, along the lens edge, and along the inner retinal edge. (**G**,**H**) Full-field scotopic ERGs (**G**) were recorded from uninjected eyes (black) and from HA-NS-500 injected eyes at various timepoints post-injection (red circles and blue squares). Line and error bars show mean ± SD. (**H**) For ERG, *n* = 10–12 injected eyes per group. * *p* < 0.05, ** *p* < 0.01, *** *p* < 0.001, and **** *p* < 0.0001 by one-way ANOVA with Tukey’s multiple comparison test. RPE: retinal pigment epithelium, ONL: outer nuclear layer, INL: inner nuclear layer, L: lens, and R: retina. Scale bars: 20 µm (**C**,**F**), 100 µm (**B**), and 200 µm (**C**,**E**,**F**).

To determine whether retinal penetration improved at later timepoints, we undertook experiments evaluating distribution of HA-NS-500 and HA-NS-250 after 1–2 weeks. HA-NS-500 were either intravitreally or subretinally injected, and animals were followed up at PI-7 days or PI-14 days with fundus imaging and tissue analysis, as in Figure 2 and Figure 3. At PI-7 and PI-14 days, intravitreally injected eyes exhibit detectable HA-NS-500 signal in fundus images (Figure 4A middle row and Figure 4D, top), suggesting the HA-NS-500 persist in the eye. The HA-NS-500 distribution pattern is more punctate and diffuse at PI-7 and PI-14 days than at the earlier timepoints. Subretinal injection (Figure 4A–D, bottom) led to a largely coherent region of HA-NS-500 in the central retina at both PI-7 and PI-14 days. Examination of retinal sections showed that at PI-7 and PI-14 days, intravitreally injected HA-NS-500 were still largely present along the ILM (white arrows, Figure 4C,E), with a small amount detectable in the retina (Figure 4C middle row, Figure 4E, top rows). In contrast, subretinal injection led to robust distribution of HA-NS-500 in the retina, specifically along the outer segment/RPE interface at both PI-7 and PI-14 days (arrowheads, Figure 4C,E, bottom row). Only autofluorescence was seen in the uninjected retina (Figure 4C, top row). A similar pattern of NS distribution was observed for HA-NS-250 (Figure 4F). At PI-14 days, HA-NS-250 was still detected in the vitreous (arrows, Figure 4F, shown at left/middle are two representative sections) with very little in the retina (arrowheads Figure 4F).

To help evaluate the safety of HA-NS-500, we conducted full-field scotopic electroretinography (ERG) prior to and at PI-7, PI-14, and PI-30 days after intravitreal or subretinal injection (Figure 4G,H). Intravitreal injection did not lead to significant changes in ERG function compared to uninjected eyes at any timepoint examined (Figure 4H, red circles). Subretinal injection is a more invasive procedure, leading to retinal detachment (observe bleb highlighted by red arrow on OCT in Figure 4B). Consistent with this tissue disruption, subretinally injected animals had significantly impaired scotopic a- and b-wave amplitudes compared to uninjected animals. This defect was most pronounced at PI-7 days, with improvement over time. By PI-30 days, scotopic a-waves had returned to baseline levels, and scotopic b-waves exhibited only minor residual impairment (Figure 4H, blue squares). These data suggest that HA-NS-500 are well tolerated in the retina, but highlight the invasive nature of subretinal injection and the need to develop effective alternative delivery strategies.

### 4.3. Sulfotyrosine Is Well-Tolerated in the Retina after Intravitreal Injection

Our initial studies demonstrated that intravitreal injection, while safe and less-invasive than subretinal injection, did not lead to appreciable penetration of HA-NS into the retina. In order to improve retinal uptake, we hypothesized that sulfotyrosine (ST) could be used to loosen the ILM and promote HA-NS uptake. Protein tyrosine sulfation is a post-translational modification that occurs in the trans-Golgi and is mediated by the enzymes sulfotransferase 1 and 2. Protein sulfation plays an important role in protein–protein interactions [29], particularly in the extracellular matrix. Sulfated proteins are highly expressed in the ILM and outer retina [30], and we hypothesized that if we disrupted protein interactions in the ILM via the delivery of free ST, ILM barrier function would be temporarily reduced, allowing improved retinal penetration of HA-NS.

**Figure 5:**
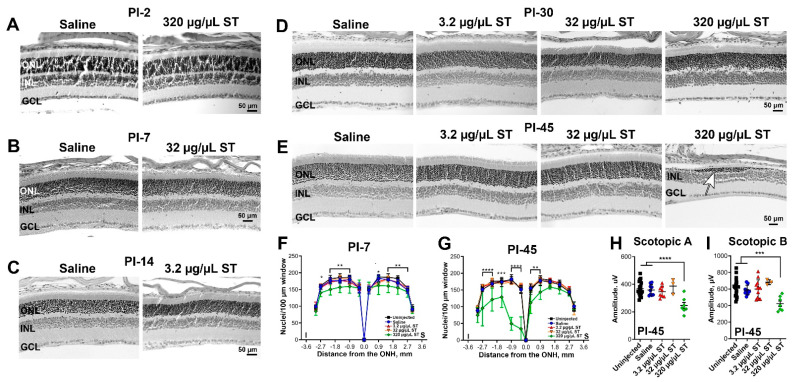
Low dose ST is safe in the retina. Adult Balb/C mice were intravitreally injected with 1.5 µL saline (vehicle) or sulfotyrosine (ST) at 3.2, 32 or 320 µg/µL. (**A**–**E**) Tissues were harvested at the indicated timepoints and retinal sections were H&E stained and imaged (original magnification 20×). White arrow highlights degenerated outer nuclear layer at PI-45 days in eyes injected with 320 µg/µL ST. (**F**,**G**) Outer nuclear layer nuclei were counted in retinal sections through the optic nerve collected at PI-7 (**F**) and PI-45 (**G**) days after ST injection. S: superior, I: inferior, and ONH: optic nerve head. Plotted is mean ± SD; * *p* < 0.05, ** *p* < 0.01, *** *p* < 0.001, and **** *p* < 0.0001 in comparison between uninjected and 320 µg/µL ST by 2-way ANOVA with Tukey’s multiple comparison test. (**H**,**I**) Full-field scotopic ERGs were recorded at PI-30 days. Plotted are mean ± SD; *** *p* < 0.001, and **** *p* < 0.0001 by one-way ANOVA with Tukey’s multiple comparison test. ONL: outer nuclear layer, INL: inner nuclear layer, GCL: ganglion cell layer, and ILM: inner limiting membrane. Scale bars: 50 µm. *n* = 3 retinas/timepoint/dose for histological analyses. *n* = 5–8 eyes/dose for ERG.

Prior to co-delivery of ST with HA-NS we evaluated whether ST led to any acute retinal toxicity. We delivered ST at three different concentrations, one (2 µL of ST at 32 µg/µL) selected to generate levels similar to the physiological concentration of tyrosine in the human vitreous [31], and two others, one ten-fold higher (2 µL at 320 µg/µL) and one ten-fold lower (2 µL at 3.2 µg/µL). Following injections, tissues were harvested at times ranging from PI-2 to PI-45 days, and retinal structure was examined. In saline-treated eyes, the ILM exhibits a normal appearance at all timepoints studied, characterized by a crisp solid line demarcating the boundary between the vitreous and the retina (Figure 5A–D). However, we observed that by PI-45 days, the eyes that received 320 µg/µL ST sometimes exhibited significant retinal degeneration, with only 1–2 rows of photoreceptor nuclei remaining in the outer nuclear layer (arrow, Figure 5E). To more fully characterize any retinal degeneration associated with ST, we counted photoreceptor nuclei across the central retina (cut along the superior–inferior plane) at PI-7 and PI-45 days to generate spidergrams (Figure 5F,G), and also examined retinal sections taken from different regions of the eye (nasal to temporal, Supplementary Figure S1). No degeneration was detected in eyes injected with the lower ST doses (3.2 µg/µL or 32 µg/µL). However, mild but statistically significant degeneration was detected in eyes injected with 320 µg/µL ST at PI-7 days (Figure 5F). By PI-45 days, there was evidence of severe outer nuclear layer thinning, specifically in the region near the site of injection (inferior central), but much less thinning outside the region of injection (Figure 5G and Figure S1). Consistent with this localized tissue degeneration, animals injected with 320 µg/µL ST also had modestly impaired scotopic a- and b-wave ERG amplitudes compared to uninjected eyes when analyzed at PI-45 days (Figure 5H,I). No ERG defect was seen in eyes intravitreally injected with 3.2 µg/µL or 32 µg/µL ST (Figure 5H,I).

To more closely evaluate the effects of ST on ILM structure, we performed ultrastructural analysis of the ILM by electron microscopy (EM) at PI-7 and PI-30 days (Supplementary Figure S2A, boxed regions are expanded views from the main image). At both timepoints, the saline treated eyes exhibit normal ILM ultrastructure characterized by tight packing of Müller glia end feet (E, Supplementary Figure S2A) with a continuous electron dense barrier line (blue arrows, Supplementary Figure S2A), and a crisply delineated, intact basal lamina (green arrows, Supplementary Figure S2A). As expected based on light microscopy, similar ILM ultrastructure was observed in eyes treated with 3.2 µg/µL and 32 µg/µL ST. Likewise, we do not observe any gross changes in ILM extracellular matrix, as indicated by intact laminin labeling in the ILM at PI-14 days (Supplementary Figure S2B).

**Figure 6:**
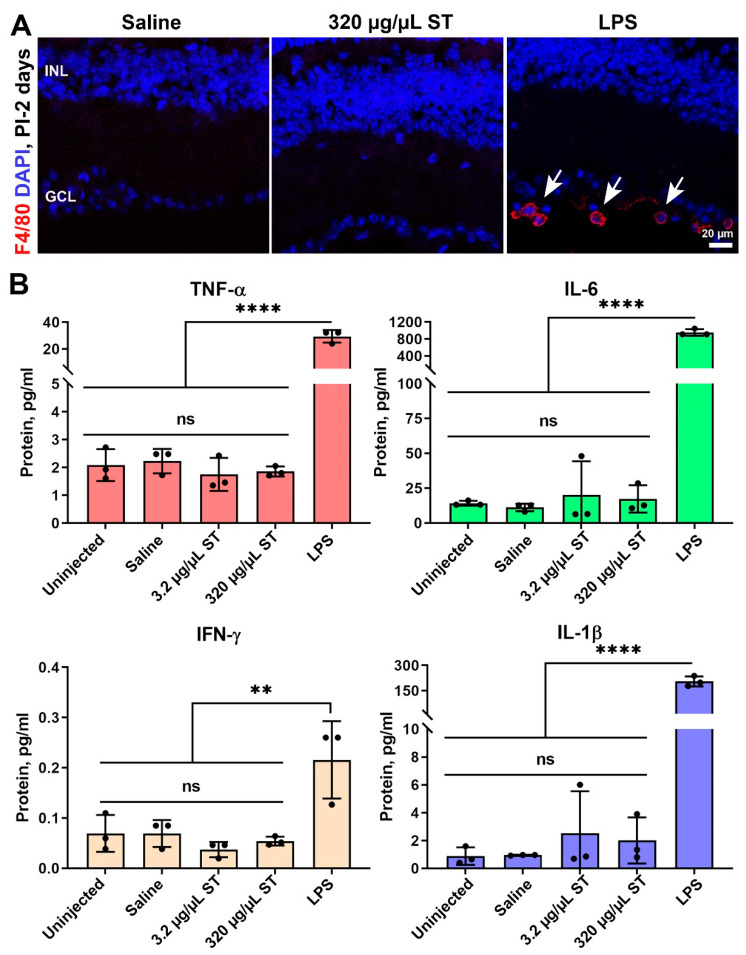
Intravitreal ST does not lead to immune response in the retina. Adult Balb/C mice were intravitreally injected with saline (vehicle) or ST at 3.2 or 320 µg/µL. (**A**) Retinal cross-sections harvested at PI-2 days were stained for the macrophage marker F4/80 (red) and nuclei (blue). Eyes intravitreally injected with 2 µL LPS at 2 µg/µL were used as a positive control. Arrows highlight F4/80 positive cells. (**B**) Whole eyes harvested at PI-2 days were pulverized and used for V-plex assay to measure cytokine protein levels. ** *p* < 0.01 and **** *p* < 0.0001 by one-way ANOVA with Tukey’s post hoc comparison. INL: inner nuclear layer, GCL: ganglion cell layer. Scale bars: 20 µm. *n* = 3 eyes per group (from separate animals) for all panels.

Low immune system activation is an essential hallmark for a prospective substance that will be used in ocular gene therapy. To examine if intravitreal injection of ST causes an acute immune reaction in the retina, we carried out immunofluorescence labeling with the macrophage marker F4/80 on frozen retinal sections collected at PI-2 days. No F4/80 positive macrophages were detected in 320 µg/µL ST-injected eyes or saline-injected eyes (Figure 6A), though many F4/80 positive cells are detected in positive control tissues injected with lipopolysaccharide (LPS). Similarly, we saw no increases in protein levels of the inflammatory cytokines TNF-α, IL-6, IFN-γ, or IL-1β at PI-2 days in eyes injected with 3.2 µg/µL or 320 µg/µL ST (Figure 6B), although robust increases were seen in LPS-injected positive controls. Combined, these data demonstrate that 3.2 µg/µL and 32µg/µL ST are well-tolerated in the eye after intravitreal delivery.

**Figure 7:**
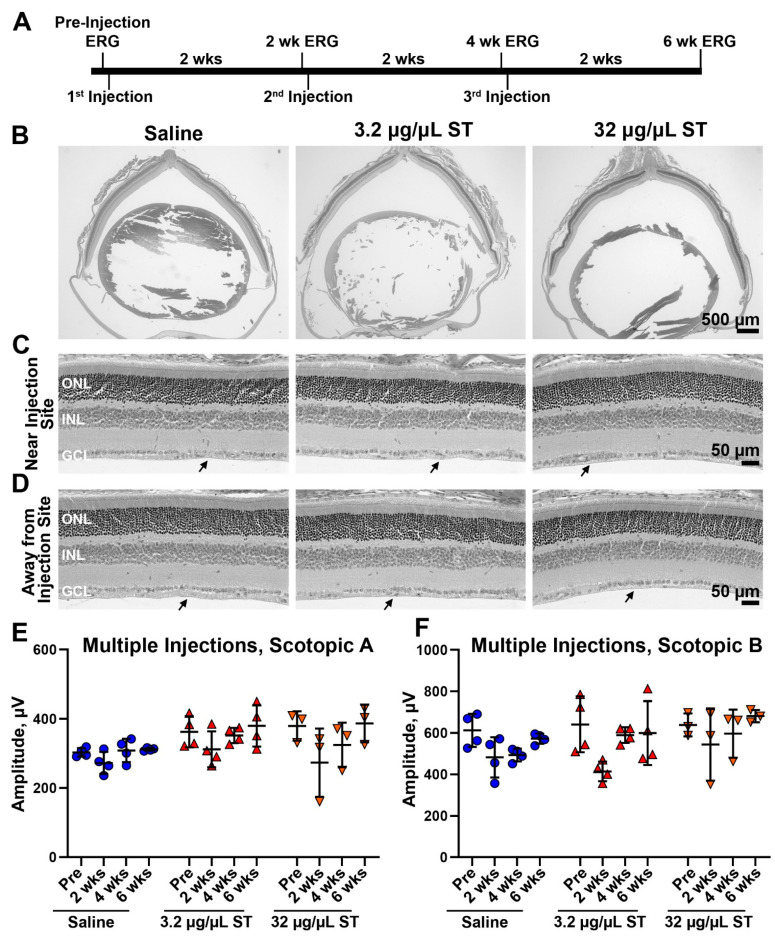
ST is well-tolerated in the retina after multiple injections. (**A**) Adult Balb/C mice were intravitreally injected with saline (vehicle) or ST at 3.2 or 32 µg/µL every two weeks as described in the schematic shown in (**A**). (**B**–**D**) Six weeks after the initial injection, tissues were harvested and processed for H&E labeling. Shown are representative retinal cross sections ((**B**), original magnification 2.5×) with magnified views both near and far from the injection site shown ((**C**,**D**), original magnification 20×). (**E**,**F**) Full-field scotopic ERGs were recorded prior to injection and every two weeks for up to six weeks. Plotted are mean ± SD. No significant differences between injected/uninjected eyes were detected by one-way ANOVA with Tukey’s multiple comparison test. ONL: outer nuclear layer, INL: inner nuclear layer, GCL: ganglion cell layer, and ILM: inner limiting membrane. Scale bars: 500 µm (**B**), 50 µm (**C**,**D**). N = 3 retinas/dose for histological analyses. *n* = 4 eyes/dose for ERG.

As the lowest two doses of ST were well-tolerated after a single injection, we next asked whether they could be safely used for multiple injections (Figure 7A). We undertook a series of experiments in which animals underwent ERG at baseline, were then injected with 2 µL ST at either 3.2 µg/µL or 32 µg/µL (or saline), and then followed up at PI-2 weeks by ERG. Immediately after the 2-week ERG was performed, eyes were re-injected with ST (second injection). Two weeks later, they again underwent ERG followed by a third injection, and two weeks after that, animals underwent a final ERG and were euthanized. This paradigm was well tolerated, with no observed retinal degeneration after three injections (Figure 7B–D) either near or away from the injection site. There were likewise no observable changes on the ILM (Figure 7C,D), and we observed no loss of ERG function at any timepoint examined (Figure 7E,F). These findings suggest that low doses (3.2 and 32 µg/µL) ST can be safely used in the eye, even after multiple injections.

### 4.4. Co-Injection of Sulfotyrosine Facilitates NS Diffusion to the Outer Retina

Our next step was to evaluate whether co-delivery of ST along with HA-NS-250 improved NS uptake in the retina. Due to the localized toxicity associated with the highest dose of ST (320 µg/µL), we proceeded using the two lower doses (3.2 and 32 µg/µL). However, before in vivo delivery, we asked whether ST would lead to aggregation of HA-NS-250 as that could influence retinal uptake. Equal volumes HA-NS-250 and ST (at 6.4 µg/µL or 64 µg/µL) were incubated together for one and a half hours at 37 °C and then fluorescently imaged. Doses of ST (3.2 µg/µL and 32 µg/µL) did not lead to aggregation of HA-NS-250 (Supplementary Figure S3).

**Figure 8:**
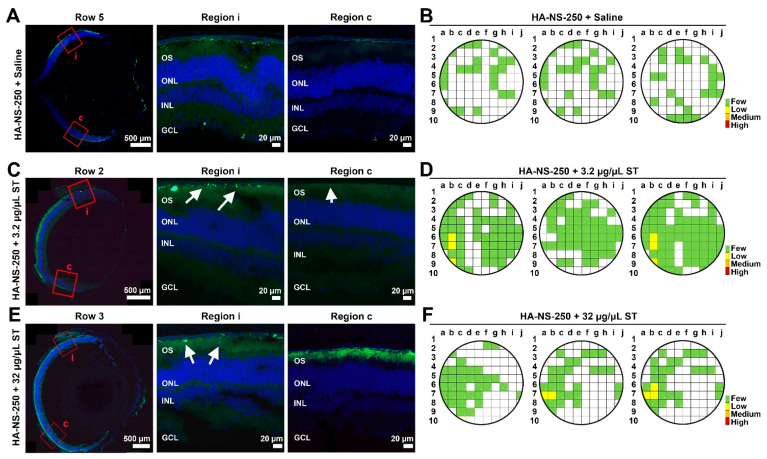
Co-injection of ST with HA-NS-250 leads to improved nanospheres uptake into the retina at PI-14 days. Adult mouse eyes were co-injected with 1.5 µL (5.8 × 10^6^ particles/µL) HA-NS-250 and either 1.5 µL saline (vehicle, **A**,**B**) or ST with a final concentration of 3.2 µg/µL ST (**C**,**D**), or 32 µg/µL ST (**E**,**F**). Tissues were collected at PI-14 days and sectioned as in Figure 2A,C,E. Shown are representative retinal cross sections (left) with red boxed regions shown on the middle and right. White arrows highlight accumulation of HA-NS-250 in the retina. (**B**,**D**,**F**) HA-NS-250 were counted in adjacent regions throughout a retinal section and in multiple sections throughout the eye. Each row corresponds to a section, and each box corresponds to a region in that section. Shown are maps from three different eyes. White squares contained fewer than 10, green squares contained 10–199, yellow squares contained 200–499, orange squares 500–999, and red squares ≥ 1000 HA-NS-250 puncta. OS: outer segments, ONL: outer nuclear layer, INL: inner nuclear layer, and GCL: ganglion cell layer. Scale bars: 500 µm and 20 µm. *n* = 3 eyes per group.

**Figure 9:**
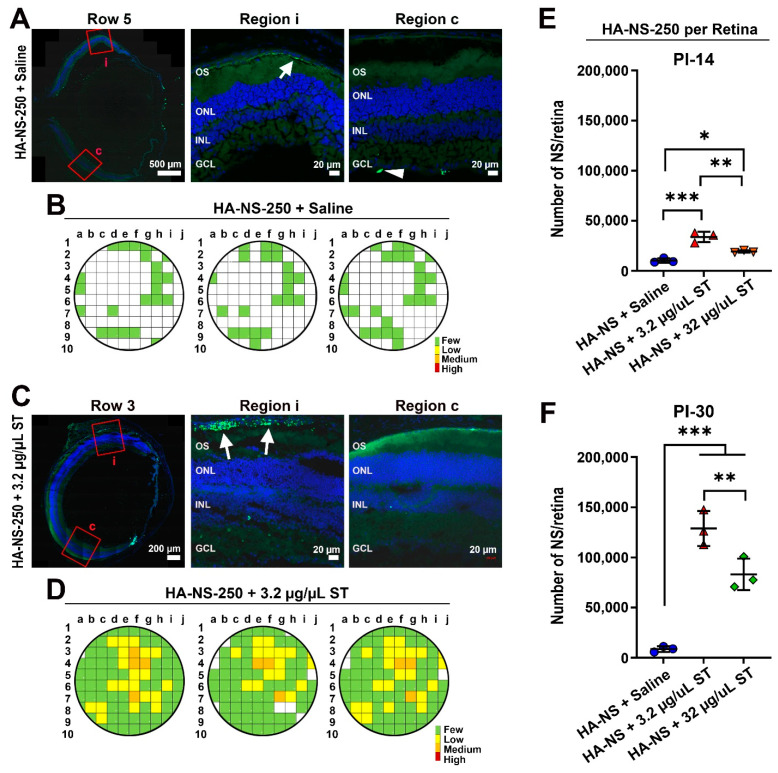
Co-injection of ST with HA-NS-250 leads to improved nanosphere uptake into the retina at PI-30 days. Adult mouse eyes were co-injected with 1.5 µL (5.8 × 10^6^ particles/µL) HA-NS-250 and either 1.5 µL saline (vehicle, (**A**,**B**)) or ST adjusted to a final concentration of 3.2 µg/µL (**C**,**D**). Tissues were collected at PI-30 days and sectioned as in Figure 2A,**C**. Shown are representative retinal cross sections (left) with red boxed regions shown on the middle and right. White arrows show accumulation of HA-NS-250 in the retina. (**B,D**) HA-NS-250 were counted in adjacent regions throughout a retinal section and in multiple sections throughout the eye. Each row corresponds to a section, and each box corresponds to a region in that section. Shown are maps from three different eyes. White squares contained fewer than 10, green squares contained 10–199, yellow squares contained 200–499, orange squares 500–999, and red squares ≥1000 HA-NS-250 puncta. (**E**,**F**) Total HA-NS-250 per retina at PI-14 and PI-30 days is plotted. * *p* < 0.05, ** *p* < 0.01, and *** *p* < 0.001 by one-way ANOVA with Tukey’s post hoc test. OS: outer segments, ONL: outer nuclear layer, INL: inner nuclear layer, and GCL: ganglion cell layer, Scale bars: 500 µm and 20 µm. *n* = 3 eyes per group.

We next asked whether co-delivery of HA-NS250 and ST led to improved uptake of NS in the retina. Eyes were intravitreally injected with 3 µL total volume containing ~8.7 × 10^6^ HA-NS-250 and either 3.2 µg/µL or 32 µg/µL ST. Tissues were harvested at PI-14 days (Figure 8 and Figure S4) or PI-30 (Figure 9 and Figure S5). As in Figure 2, unfixed eyes were sectioned, and sections were taken from throughout the eye. Adjacent images were captured from each section and HA-NS-250 fluorescent puncta in the retina (fluorescence in the vitreous was not counted) were counted in each image to generate semi-quantitative graphical maps of HA-NS-250 distribution. As expected based on experiments described in Figure 4, at PI-14 days, very little retinal HA-NS-250 is detected in eyes injected with HA-NS-250 alone (Figure 8A and Figure S4A show representative images and Figure 8B shows graphical maps from three example eyes). However, robust HA-NS-250 fluorescence is detected at PI-14 days in eyes injected with HA-NS-250 and 3.2 µg/µL ST (Figure 8C,D and Figure S4B). The signal is largely localized to the outer retina (arrows, Figure 8C), and fairly well distributed throughout the eye, although some regions still lack appreciable HA-NS-250 accumulation. Results at PI-14 days using HA-NS-250 and 32 µg/µL ST (Figure 8E,F and Figure S4C) were in between those seen with no ST and those seen with the 3.2 µg/µL ST. We observed more HA-NS-250 in the retina (arrows, Figure 8E) compared to eyes not receiving ST, but the distribution of HA-NS-250 in the eye after co-treatment with 32 µg/µL ST was not as robust as after co-treatment with 3.2 µg/µL ST (Figure 8F vs. Figure 8D).

As we obtained good results with the 3.2 µg/µL dose of ST at PI-14 days, we followed up another group treated with this paradigm. At PI-30 days, intravitreal injection of 3.2 µg/µL ST coupled with HA-NS-250 led to much more robust accumulation of HA-NS-250 in the retina than in eyes that did not receive ST (Figure 9C,D vs. Figure 9A,B). Similar to PI-14, at PI-30 most of the HA-NS-250 in eyes co-injected with 3.2 µg/µL ST was found in the outer retina at the RPE/outer segment interface (white arrows Figure 9C).

To achieve a better comparison between groups and timepoints, we estimated the total number of HA-NS-250 in the retina (Figure 9E,F) based on the number counted in each section and the estimated total number of sections in the retina. At both PI-14 and PI-30, eyes co-injected with 3.2 µg/µL ST had more HA-NS-250 in the retina than other groups. Interestingly, in eyes co-injected with 3.2 µg/µL ST, the number of HA-NS-250 fluorescent puncta in the retina went up dramatically between PI-14 and PI-30 days (Figure 9E vs. Figure 9F), suggesting that there is continued uptake from the vitreous over time when low doses of ST are delivered along with the HA-NS-250. This increase between PI-14 and PI-30 days was not seen in eyes that did not receive ST with the HA-NS-250. Finally, we confirmed that co-delivery of HA-NS-250 and ST did not elicit any defects in retinal function, by performing ERG before injection and at PI-30 days (Supplementary Figure S5C). Combined, these data show that low levels of ST (3.2 µg/µL) lead to dramatic improvements in retinal penetration and accumulation of HA-NS-250 without any acute retinal toxicity.

## 5. Discussion

Here, we implement an innovative approach to help generate persistent outer retinal delivery of nanocarriers after intravitreal injection. We utilize biocompatible photo-cross-linked HA-NS, and pair this formulation approach with delivery of ST to transiently open the ILM. As the HA-NS are highly cross-linked in either a 250 nm or 500 nm size, they do not rapidly degrade and persist well in the vitreous over the course of several weeks and along the ILM; however, they are not readily taken up into the retina. On the other hand, when paired with low-dose ST, HA-NS-250 exhibit robust sustained uptake into the outer retina over the course of the study (30 days). We demonstrate that HA-NS and low-dose ST are safe for use in the eye, do not elicit an acute immune response, and do not lead to retinal toxicity (i.e., retinal degeneration or decreased ERG responses).

HA, a hydrophilic negatively charged polymer, has been widely used as an important component of diverse carrier systems for diagnostics and therapeutics including for the treatment of posterior eye diseases [7]. It attracted research attention due to its ability to enhance gene delivery and expression in the retina [32]. It is often used as a coating for lipid- or carbohydrate-based drug and gene carriers for retinal delivery [33,34], and has been shown to promote mobility within the vitreous [35]. These biochemical features of HA are likely what contribute to its ability to persist in the outer retina and lead to sustained uptake over time. Although here we analyze HA-NS uptake for only the first month, HA-NS remained in the outer retina at PI-30 days, and we look forward to examining longer timepoints in future studies. Another key feature of HA-based NS is that they are biodegradable. HA is a critical component of the normal vitreous and mechanisms that lead to turnover of HA (including digestion by hyaluronidases and other oligosaccharide targeting enzymes), also likely leading to the slow degradation of HA-NS in the vitreous, preventing them from permanently accumulating in the vitreous.

Here, our goal was to evaluate retinal penetration of HA-NS; however, a critical next step for future studies will be assessing uptake into outer retinal cells such as photoreceptors and the RPE. HA mediates cellular uptake through interactions with the CD44 receptor [33,36,37] which has been shown to promote internalization of HA-coated nanoparticles into the RPE [38,39]. CD44 expression has been reported in Müller glia and in several retinal neuronal cell populations, including some amacrine and ganglion cells [40,41,42]. However, there are also data from other tissues indicating that HA can be taken up into cells in a CD44-independent manner [43], suggesting that CD44 expression may not be a requirement for cellular uptake in the retina. In addition, studies suggest that HA can help promote gene expression after particles are taken into the cell, for example by promoting an intracellular trafficking pathway that avoids lysosomal components and leads to accumulation in the perinuclear region [43,44], and by potentially enhancing transcriptional activity on transfected DNA [45]. Our ultimate goal is to use these HA-NS to deliver genetic material, and though here we utilize HA-NS with an attached fluorescent reporter dye, HA can be conjugated to nucleic acids [46].

However, in spite of the manifold uses and benefits of HA-conjugated particles, they have been reported to have difficulty crossing the ILM [35], a finding we recapitulated in our initial studies here. To help overcome this barrier, we introduce ST. Protein-tyrosine sulfation plays a significant role in protein–protein interaction. High levels of tyrosine-sulfated proteins are found in the ILM [30], and ablation of any of the enzymes responsible for this modification did not compromise the ILM structurally [47]. As the tyrosine sulfation has been shown to be involved in protein–protein interactions [48], we hypothesized that co-delivery of ST and HA-NS would lead to disruptions in protein–protein interactions in the extracellular matrix of the ILM, thus leading to a transient reduction in ILM barrier function and increased retinal permeability. We identified a dose of ST (3.2 µg/µL) that elicited no retinal toxicity but promoted penetration through the ILM. This is important, as our goal is not to eliminate or degrade the ILM, but rather temporarily reduce its barrier function. Co-delivery of ST had the desired functional benefits, resulting in dramatically increased uptake of HA-NS into the outer retina. While we did observe some HA-NS in the inner retinal layers, after delivery with ST, most retinal HA-NS-250 localized to the outer retina between the photoreceptors and the RPE, the ideal region for targeting most IRDs.

In conclusion, we here present data on a novel and highly efficacious method to improve retinal penetration of biocompatible nanocarriers after intravitreal delivery. Enhancing the efficacy of intravitreal delivery is a key therapeutic goal for both the treatment of IRD and other forms of retinal degeneration that affect the outer retina, so these findings represent a significant advancement. We look forward to utilizing this approach in future to evaluate gene delivery in preclinical models, and hope that effective clinical intravitreal delivery to the outer retina will soon be within reach.

## Data Availability

Data are contained within the article.

## Supplementary Materials

The following are available online at https://www.mdpi.com/article/10.3390/pharmaceutics13091510/s1, Figure S1: Retinal thinning in response to high-dose ST is limited to the region of injection, Figure S2: Sulfotyrosine does not elicit ultrastructural changes in the ILM, Figure S3: ST at low doses does not induce aggregation of HA-NS-250, Figure S4: Co-injection of ST with HA-NS-250 leads to improved nanosphere uptake into the retina at PI-14 days, Figure S5: Co-injection of ST with HA-NS-250 leads to improved nanospheres uptake into the retina at PI-30 days.
