# Supplementary material for: Co-Injection of Sulfotyrosine Facilitates Retinal Uptake of Hyaluronic Acid Nanospheres Following Intravitreal Injection
Source: Pharmaceutics. 2021 Sep 18;13(9):1510. doi: 10.3390/pharmaceutics13091510 (PMC8469555; doi:10.3390/pharmaceutics13091510)
Supplement: Supplementary file 1 [file pharmaceutics-13-01510-s001.zip › pharmaceutics-1355441-supplementary.pdf]

pharmaceutics

Supplementary Materials: Co-Injection of Sulfotyrosine Facilitates Retinal Uptake of Hyaluronic Acid Nanospheres Following Intravitreal Injection

Aiden Eblimit, Mustafa S. Makia, Daniel Strayve, Ryan Crane, Shannon M. Conley, Tirthankar Sinha, Ghanashyam Acharya, Muayyad R. Al-Ubaidi and Muna I. Naash

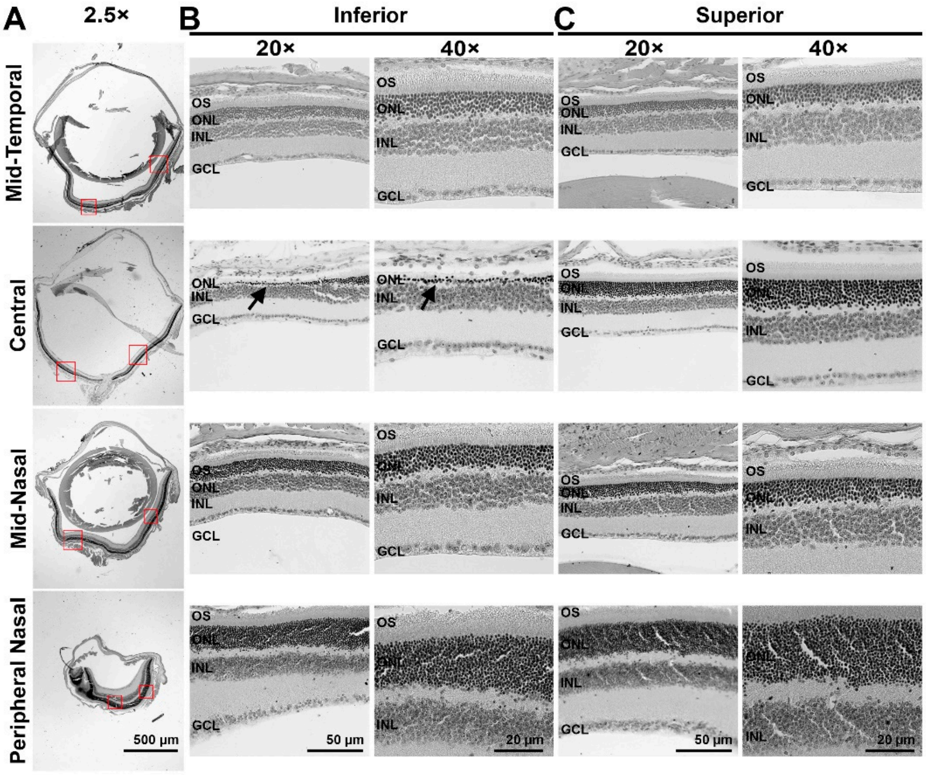

Figure S1. Retinal thinning in response to high-dose ST is limited to the region of injection. Adult Balb/C mice were intravitreally injected with 320 μg/μL ST in the inferior central region of the retina. At PI-45 days, tissues were harvested, sectioned along the inferior-superior plane, and processed for H&E labeling. Each row shows a representative section from a different region of the retina. Red boxes in (**A)** (original magnification 2.5×) reflect areas that are shown at higher magnification in (**B)** (inferior region, original magnification 20× and 40×) and (**C)** (superior region, original magnification 20× and 40×). Black arrows highlight ONL thinning in the inferior central retina. ONL: outer nuclear layer, INL: inner nuclear layer, GCL, ganglion cell layer, OS: outer segment layer. Scale bars: 500 μm (**A**), 50 μm (**B,C**), and 20 μm (**B,C**).

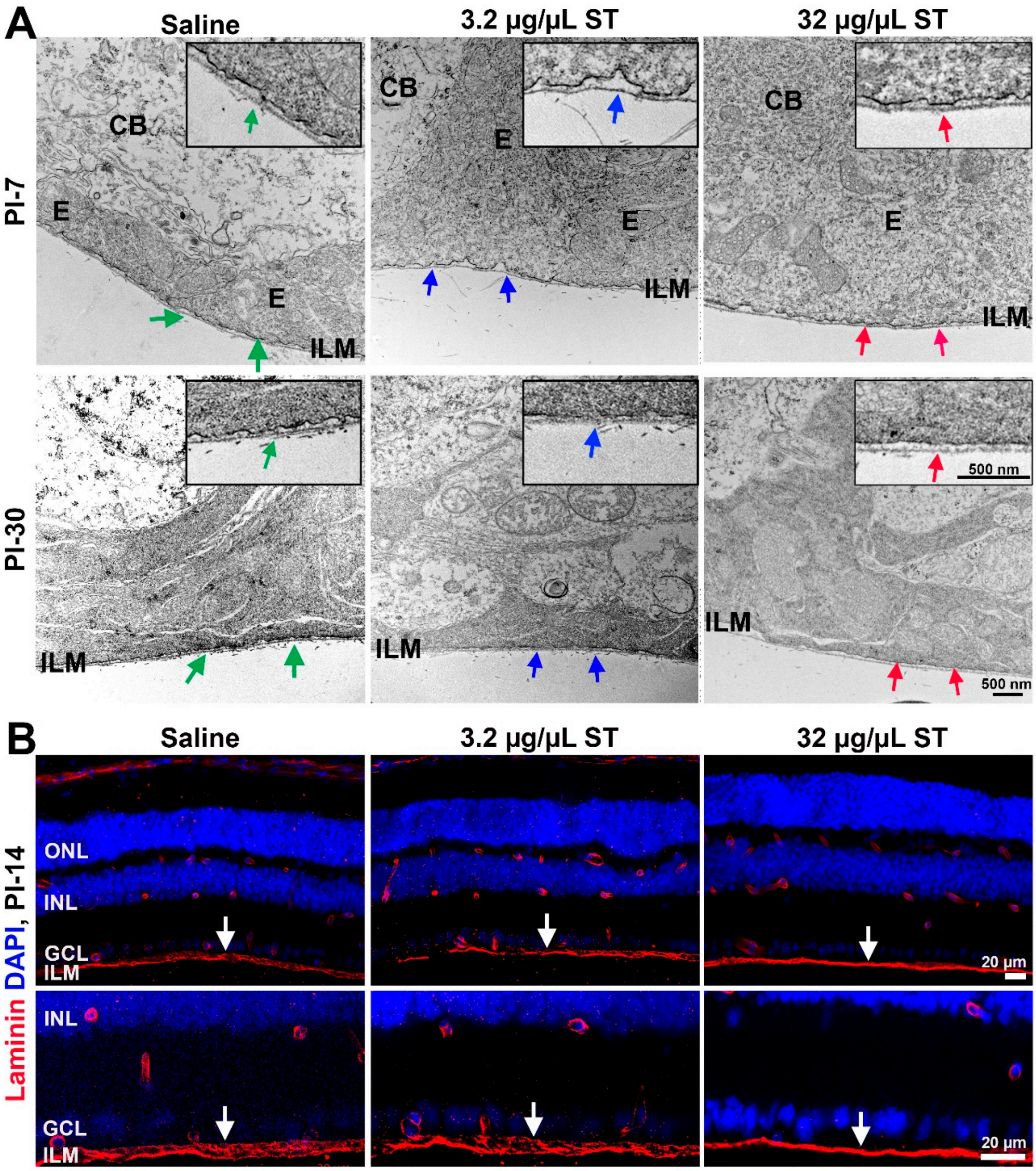

Figure S2. Sulfotyrosine does not elicit ultrastructural changes in the ILM**.** Adult mice were intravitreally injected with ST at the indicated doses and tissues were collected at PI-7 and PI-30 days and processed for EM (**A**) or immunofluorescence (**B**). (**A)** Shown are representative transmission EM images of the ILM and adjacent area. Blue arrows highlight intact electrodense line along the inner edge of the ILM. Green arrows highlight intact, crisply delineated basal lamina. *n* = 3 eyes/group, captured at 25,000×. (**B)** Retinal sections were labeled for the ILM component laminin (red). Nuclei are counterstained with DAPI (blue). Bottom panels show magnified view (captured at 40×) of the ILM from full images shown on top (captured at 20×). ONL: outer nuclear layer, INL: inner nuclear layer, GCL: ganglion cell layer, ILM: inner limiting membrane. E: astrocyte endfeet, CB: neuronal cell body Scale bars: 500 nm (**A**), 20 µm (**B**).

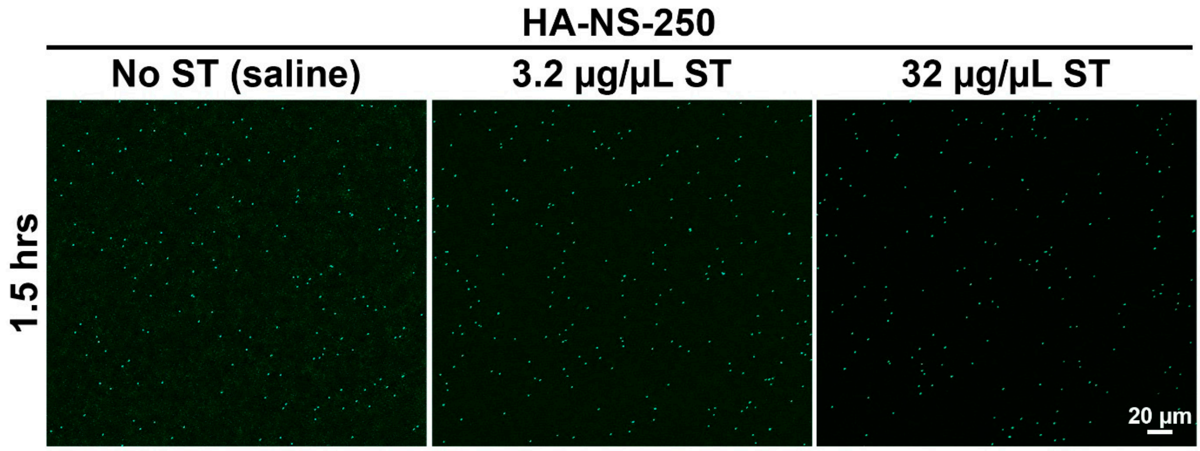

Figure S3. ST at low doses does not induce aggregation of HA-NS-250. HA-NS-250 (5.8×10^6 HA-NS-250/µL) was incubated for 1.5 hours with equal volumes of saline (vehicle) or ST at 6.4 µg/µL, or 64 µg/µL, prior to being diluted and imaged under a microscope for native fluorescein fluorescence. Scale bar: 20 µm.

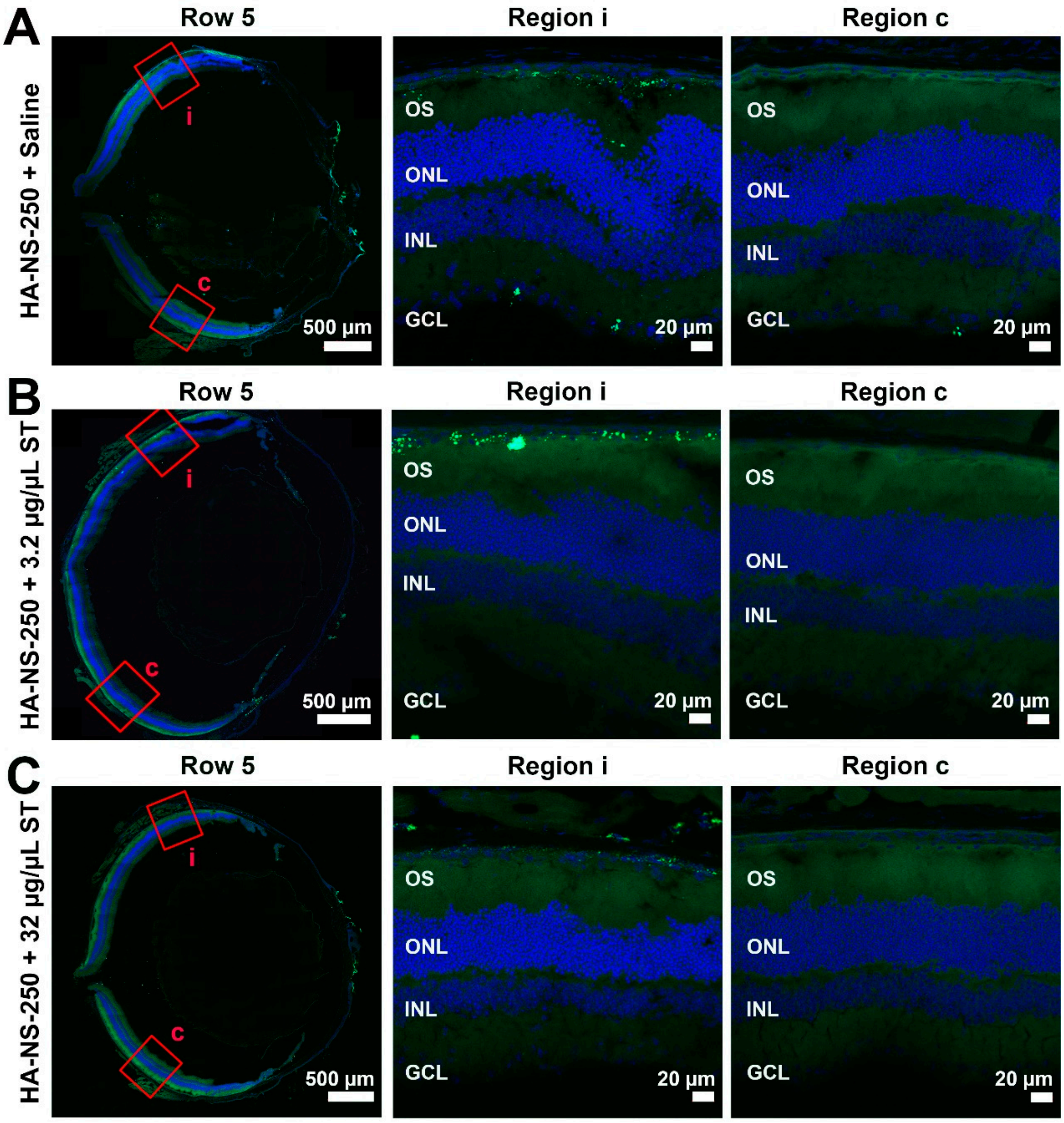

Figure S4. Co-injection of ST with HA-NS-250 leads to improved nanosphere uptake into the retina at PI-14 days. Shown are additional representative images from the eyes shown in Figure 8**.** Adult mouse eyes were co-injected with 1.5 µL (5.8 × 10^6 particles/µL) HA-NS-250 and either 1.5 µL saline (vehicle, **A**) or ST adjusted to a final concentration of 3.2 µg/µL (**B**), or 32 µg/µL ST (**C**). Tissues were collected at PI-14 days and sectioned. Shown are representative retinal cross sections (left) with red boxed regions shown on the middle and right. OS: outer segments, ONL: outer nuclear layer, INL: inner nuclear layer, GCL: ganglion cell layer, Scale bars: 500 µm and 20 µm.

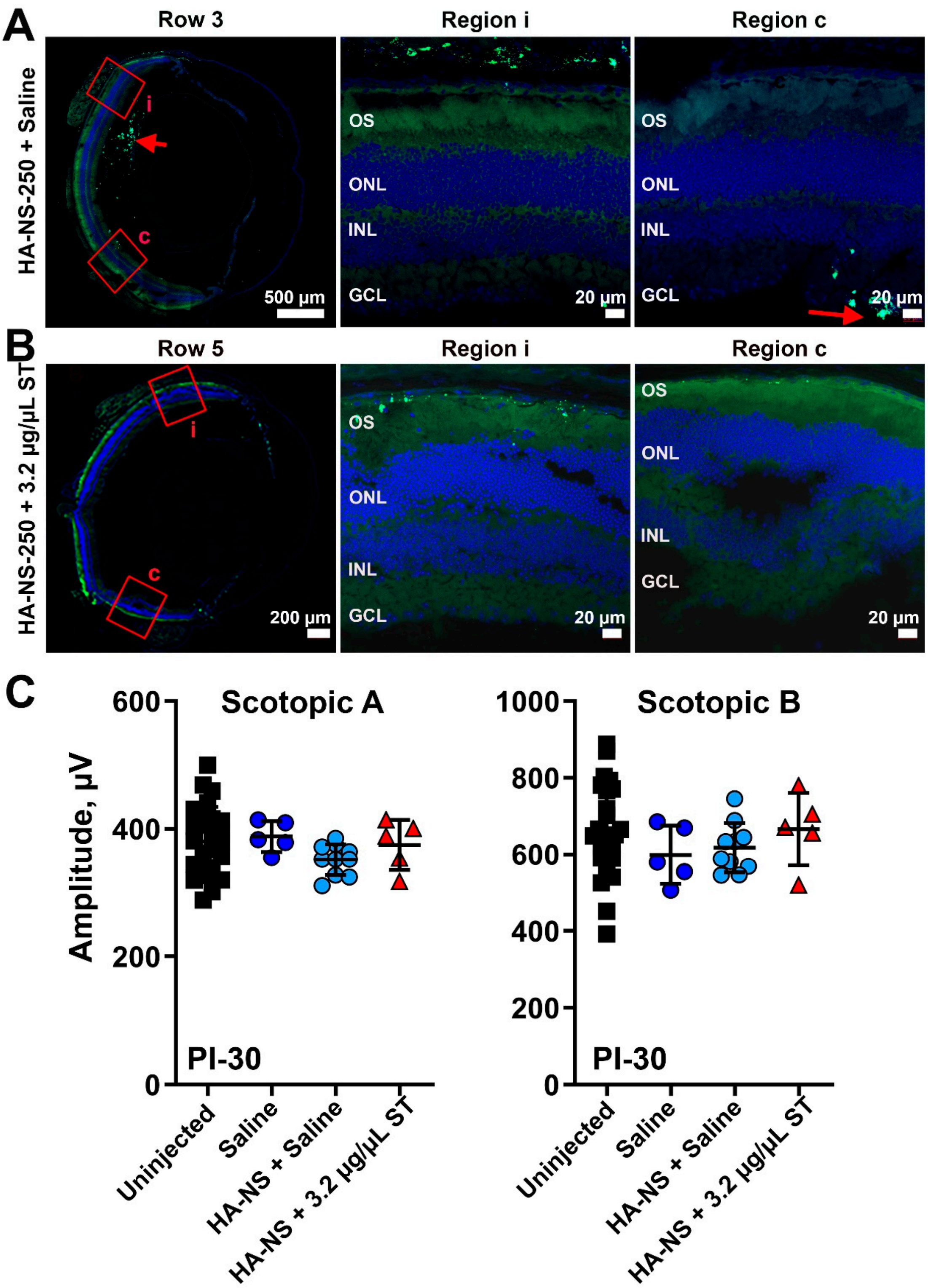

Figure S5. Co-injection of ST with HA-NS-250 leads to improved nanospheres uptake into the retina at PI-30 days. Shown are additional representative images from the eyes shown in Figure 9. Adult mouse eyes were co-injected with 1.5 µL (5.8 $\times 10^6$ particles/µL) HA-NS-250 and either 1.5 µl saline (vehicle, **A**) or ST adjusted to a final concentration of 3.2 µg/µL (**B**). Tissues were collected at PI-30 days and sectioned. Shown are representative retinal cross sections (left) with red boxed regions shown on the middle and right. Red arrows highlight HA-NS-250 in the vitreous. **C.** Full-field scotopic ERGs were recorded at PI-30 days. No significant differences between groups were detected by one-way ANOVA with Tukey's multiple comparison test. OS: outer segments, ONL: outer nuclear layer, INL: inner nuclear layer, GCL: ganglion cell layer, Scale bars: 200 µm and 20 µm.
